# Aquaculture Production and Value Chains in the COVID-19 Pandemic

**DOI:** 10.1007/s40572-022-00364-6

**Published:** 2022-06-17

**Authors:** Nesar Ahmed, Mohamad N. Azra

**Affiliations:** 1 School of Life and Environmental Sciences, Deakin University, Burwood, VIC 3125 Australia; 2 Climate Change Adaptation Laboratory, Institute of Marine Biotechnology, Universiti Malaysia Terengganu, 21030 Kuala Terengganu, Malaysia

**Keywords:** Aquaculture, Pandemic, Seafood, Supply chain, Consumption

## Abstract

**Purpose of Review:**

The purpose of this review is to summarize the impacts of the coronavirus disease 2019 (COVID-19) pandemic on aquaculture input supply, production, distribution, and consumption.

**Recent Findings:**

The COVID-19 pandemic–related lockdowns, social distancing, supply chain disruptions, and transport restrictions affect seafood production, distribution, marketing, and consumption. Recommendations are suggested to overcome these challenges.

**Summary:**

The COVID-19 has led to disruption of aquaculture practices worldwide. The pandemic has adversely affected the aquaculture input supply of fish stocking and feeding, which, in turn, has impacted aquaculture production. Moreover, the COVID-19 crisis has had adverse effects on value addition to aquaculture products, through the restrictions of seafood marketing and exporting. Aquatic food production is vulnerable to the effects of COVID-19 outbreak; hence, adaptation strategies must be developed to cope with the challenges. There is an urgent need for collaboration among key stakeholders to rebuild the supply chain of inputs and fish marketing for sustainable aquaculture practices. International agencies, donors, government and non-governmental organizations, researchers, and policymakers need to develop policies to support aquaculture production and supply chains.

## Introduction

The novel coronavirus disease 2019 (COVID-19) is a current global challenge [1–3]. Like other food-producing sectors, COVID-19 hits aquatic food production worldwide. In fact, the COVID-19 pandemic with its subsequent lockdowns and restrictions is having adverse effects on aquaculture practices and production [4••, 5•, 6, 7••]. Aquaculture is the human cultivation of aquatic organisms in waters [8], and aquaculture operation is not possible without direct human involvement. Different aquaculture practices are the outcomes of human–environment interactions, as humans have created aquaculture through the manipulation of freshwater, brackish water, and marine habitats.

Aquaculture is the fastest growing food production sector in the world, with an average annual growth rate of 5.3% during 2001–2018 [9]. Global aquaculture production reached 82.1 million tons in 2018, of which inland aquaculture produced 51.3 million tons (62%), while both coastal and marine aquaculture yielded 30.8 million tons (38%) [9]. Because of favorable environmental and climatic conditions, aquaculture has developed strongly in tropical and subtropical regions, but it is also practiced in temperate region. Asia contributes 89% to global aquaculture production. Among aquaculture-producing countries in the world, China is ranked first (58% of total production), followed by India, Indonesia, Vietnam, Bangladesh, Egypt, Norway, Chile, Myanmar, and Thailand [9].

Globally, common aquaculture practices are cage culture, floodplain aquaculture, net-pen culture, pond aquaculture, raceway farming (flow-through system), raft/long-line culture, recirculating aquaculture systems, and rice-fish farming. These farming practices can be classified into single-species monoculture, multi-species polyculture, and integrated farming [10]. Based on farming inputs (fish fry and feed), aquaculture can be grouped into extensive, semi-intensive, and intensive production [11]. Extensive production typically uses slightly modified versions of traditional methods with low input while a semi-intensive method applies higher inputs, but lower than intensive farming [10]. Although a total of 622 aquatic species being recorded in aquaculture worldwide, most dominant species are carp, catfish, shrimp, salmon, and tilapia. Globally, salmon (with trout) is the most traded fish product in terms of value followed by shrimp (with prawn), while carp is the foremost group of aquaculture in terms of volume [9].

The COVID-19 pandemic is a leading challenge to aquaculture practices worldwide that has significantly affected the production of seafood, which refers to all freshwater, brackish water, and saltwater fish including crustacean and shellfish. Because of COVID-19 impacts, global aquaculture output drops for the first time in 60 years [12]. As the pandemic is still unfolding with new variants (e.g., Alpha, Beta, Gamma, Delta, Lambda, and Omicron) have emerged, the impact of COVID-19 could have even more dramatic, unforeseen, and longer-term effects on global aquaculture production. It seems that further impacts and consequences await in terms of aquaculture practices by the COVID-19 pandemic. If we could not tackle this situation properly, aquaculture production may be uncertain for next few years. Considering the vulnerability of aquaculture production to the effects of COVID-19 pandemic, adaptation strategies must be developed to cope with the challenges.

During the global COVID-19 pandemic period, fish as well as seafood remains a good source of essential nutrients for the human diet, with protein of high biological value, human health-promoting essential omega-3 fatty acids, and trace elements [13, 14, 15••]. In fact, seafood consumption provides nutrients linked to reductions in hunger, malnutrition, and disease for global population [16, 17]. Fish support about 3.3 billion people worldwide with 20% of their animal protein consumption, and per capita fish intake has risen from 9 kg in 1961 to 20.5 kg in 2018, on average fish consumption increase 3.2% per annum, which is twice of global annual population growth rate (1.6%) for the same time [9]. To meet the project demand of population growth, global aquaculture production need to reach 109 million tons by 2030 [9] and 140 million tons by 2050 [18]. Thus, global aquaculture production needs to continue to supply protein and nutrients for the human diets during and after the pandemic.

This paper reviews the impacts of COVID-19 on aquaculture production and value chains. The aim of this article is to provide a global snapshot about key challenges of aquaculture production during the pandemic and to provide suggestions for adaptation to the pandemic. We consider potential impacts using examples from published papers, reports, and media coverage from March 2020 to January 2022. We discuss the effects of COVID-19 on aquaculture production in terms of supply-driven input- and demand-driven output-related marketing constraints during the pandemic.

## Aquaculture Practices in the COVID-19 Pandemic

**Fig. 1:**
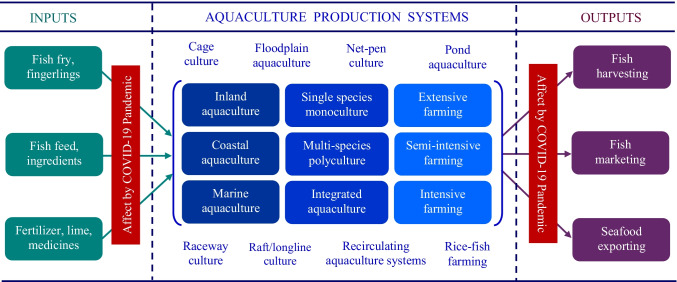
The COVID-19 pandemic has affected the input supply of aquaculture practices and output marketing

Although COVID-19 does not infect aquatic species [19], the pandemic has disrupted, or at least slow down, most aquaculture practices globally [4••, 7••]. A significant number of farmers had to temporarily stop fish production or severely reduced their aquaculture practices during the pandemic [20, 21]. COVID-19 is now a global health crisis for humans, and thus, human–environment interactions in aquaculture are vulnerable to the COVID-19 pandemic, as land-based aquaculture is widely exposed to pandemic fears [4••]. The pandemic has affected various aquaculture practices in the world. In fact, aquaculture is highly susceptible to the pandemic that has led a number of challenges to input supply, and then is subsequently impacting grow-out operations. The COVID-19 pandemic and subsequent transport restrictions, lockdowns, and physical distancing have all affected aquaculture practices, in the first instance, by limiting input supply [4••, 7••, 22]. Inadequate and irregular supply of inputs during the lockdown period has compromised aquaculture operation, resulting in a jeopardized output of aquaculture products, as well as seafood marketing challenges (Fig. 1).

COVID-19–related lockdowns and restrictions have impacted global aquaculture production. The latest estimates of global aquaculture production in 2020 suggest a drop of nearly 2% due to the COVID-19 pandemic [23]. The damage to the aquaculture sector in China caused by COVID-19 has been reported to be far-reaching due to input, production, marketing, and export problems [24]. It has been reported that COVID-19 has adverse impacts on aquaculture production in a number of Asian countries, including Bangladesh, Cambodia, India, Indonesia, Laos, Myanmar, the Philippines, Thailand, and Vietnam [21, 25–27]. In Malaysia, aquaculture production has been affected by the pandemic due to transport disruption, interruption of supply chains, reduction in feed supply, and reduction in seafood demand [20, 28]. Aquaculture in Latin America and Caribbean has also been reported to have been negatively affected by the COVID-19 crisis [29]. The Australian, European Union (EU), United Kingdom (UK), and United States of America (USA) aquaculture industries, as well as seafood production, have all been experiencing major disruptions during the pandemic [30–33].

Globally, pangasius catfish production is expected to decrease of about 7% in 2020 due to the pandemic [34]. Because of low stocking and slow feeding in pangasius aquaculture during the pandemic, farming area decreased by 26% in the Mekong Delta, which, in turn, is expected to lead to a drop of around 1.2 million tons of total output in 2020 [34]. The growth of global salmon production may be slow to 2–4% due to the pandemic [35]. The effects of the COVID-19 pandemic continue to be the primary concern of the farmed salmon industry in Chile [23]. Salmon farming in Chile has also been affected as a result of limited access to farms, inadequate processing capacity, and reduced market demand [36]. Finfish and shellfish production has already been affected by COVID-19–related lockdowns and restrictions [37, 38•]. Globally, shrimp production has been disrupted by the pandemic due to the interruption of farming practices [4••]. In India, an economic loss of US$1.5 billion was estimated to the shrimp sector by the pandemic in 2020 [38•]. The shrimp industry of Iran has also been affected by COVID-19 due to adverse impacts on hatchery production, limited post-larval stocking, lower production, and marketing constraints [39].

The pandemic with anthropogenic stressors will represent considerable economic challenges to aquaculture systems across the world [40]. In some cases, COVID-19 has combined the effects of simultaneous stressors that can make the concern of seafood supply, including climate change, natural hazards, and war [15••]. There are cumulative impacts of COVID-19 and climate change on marine aquaculture [41]. Moreover, COVID-19, cyclones, and monsoon flooding affect coastal aquaculture [42, 43]. There has been a coincidence of climate change, COVID-19, fuel price increases, and war (e.g., Russia, Ukraine, Yemen)—all of these factors may affect aquaculture in the future [15••, 44].

## Input Supply Disruption

**Fig. 2:**
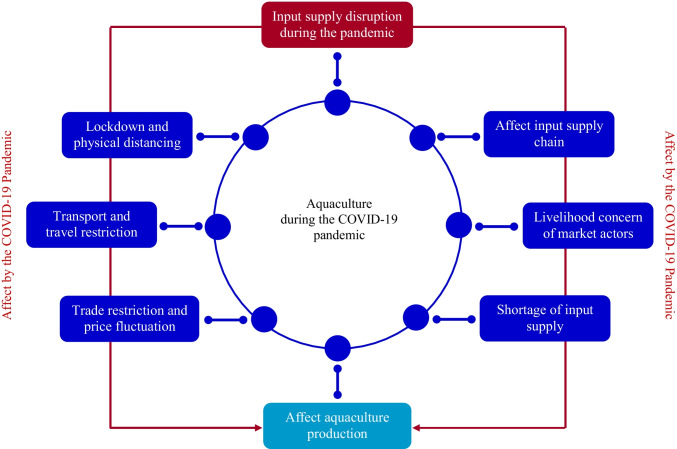
COVID-19 disruptions and impacts on input supply, which, in turn, affect aquaculture production

The protection measures against the spread of COVID-19 taken by governments of almost all countries across the world have directly affected each step of aquaculture supply chains [5•]. In fact, the COVID-19 crisis is having a series of adverse impacts on aquaculture input supply chains. Ultimately, the pandemic lockdowns with domestic and international transport interruptions and travel restrictions have led to reduce capacity in aquaculture input supply. There is great cause of concern about the impact of COVID-19 pandemic on input supply of various aquaculture practices. Because of inadequate and/or irregular input supply, COVID-19 affects aquaculture in several Asian countries, including Bangladesh, China, India, Indonesia, Myanmar, the Philippines, Thailand, and Vietnam [21, 27]. There are concerns of aquaculture practices by limiting input supply, which undermine fish production (Fig. 2). Aquaculture production capacity will be largely affected by COVID-19 pandemic–related input supply disruptions [45]. Thus, it can be expected that aquaculture production will vary greatly with variation of input supply (Table 1). Sourcing alternative inputs may help to adapt the challenges of COVID-19 [44].

**Table 1:** Aquaculture input- and output-related concerns during the COVID-19 pandemic

Aquaculture	Item	Element	COVID-19–related concern	Impact	Outcome
Input	Seed	Fish fry/fingerlings, prawn and shrimp post-larvae/juveniles	Inadequate broodstock supply, affected hatchery production, reduced wild fry collection	Irregular fry supply, poor quality fry, affected fish stocking	Uncertainty of aquaculture productivity
	Feed	Industrially manufactured pelleted feed, farm-made feed, and supplementary feed	Inadequate supply of feed ingredients, less availability and access to feed ingredients, affected feed manufacturing	Varying feed production, low-quality feed, affected feeding of fish	
	Other input	Fertilizer, growth hormone, lime, antibiotics, and probiotics	Interruption of production and distribution, supply disruption, less availability	Irregular application, poor water quality, concern of fish health management	
Output	Harvesting	Fish harvesting, landing, and distribution	Not harvesting timely, disruption of fish distribution, unable to sell fish	Varying fish availability, affected consumption, change in consumer behavior	Uncertainty of economic profitability
	Marketing	Post-harvest handling, fish supply, transportation, and trading	Unable fish transportation, mobility restriction of market actors, cutoff marketing channel	Price variation, trade restriction, closure of fish markets	
	Exporting	Seafood processing, packaging, and shipment	Shutdown processing plants, international border restriction, shipment cancellation	Decline seafood export, reduced seafood demand, international market disruption	

### Concern of Fish Stocking

Aquaculture is primarily dependent upon an adequate supply of fish stocking, which is the first input of grow-out operation. Globally, most aquaculture practices depend on hatchery-produced fry. Fish stocking depends on a number of factors, including the selection of species, size of stocked fish, number of fish, and farming methods. Single species of fish are stocked in monoculture (e.g., salmon farming, shrimp culture) while different species are stocked in polyculture (e.g., carp polyculture, carp-tilapia farming). Based on size, fish are stocked as fry, fingerling, and juvenile, depending on availability, stocking season, culture duration, and methods. There is great potential to increase fish productivity through an increase in fish stocking densities at certain level.

However, one of the major bottlenecks for the stocking of hatchery-produced fish in grow-out operation is the inadequate supply of quality fry due to the pandemic. Moreover, increased prices of fish fry as a result of limited supply have affected aquaculture grow-out operation in many developing countries [26, 46]. In order to produce quality fish fry, hatchery operation for some target species often depends on wild-caught broodstock (i.e., mother fish or berried females). In fish hatcheries, broodstock are essential component for continuous operation. However, hatchery operators cannot get good quality wild broodstock due to transport restrictions, which is usually transported from natural water resources with long distance. During the COVID-19 pandemic, a number of fish hatcheries in aquaculture-producing countries were unable to manage broodstock [21, 37]. For example, shrimp hatcheries in India, Iran, and Thailand were seriously affected by inadequate supply of broodstock [37, 38•, 39]. In Bangladesh, some hatcheries were closed due to lack of broodstock [21]. Thus, the quality of hatchery fry remains a concern due to inadequate and irregular supply of broodstock.

Fish hatchery operation has been temporarily stopped in many developing countries during the pandemic [47]. Broadly, COVID-19 outbreak has adversely affected hatchery production with selling of fry in several aquaculture-producing countries, including Bangladesh, Egypt, India, Myanmar, Nigeria, and Timor-Leste [47, 48•]. A number of fish hatcheries in Bangladesh have been closed during the pandemic because of unable to manage pituitary gland hormones for hatchery operation [21]. In general, pituitary gland hormones for hatchery operation are mostly imported from India [49], which is impeded due to lockdowns and transport restrictions. Moreover, there has been a risk of hatchery management due to a delivery problem of liquid oxygen [50].

The transportation of fry from hatcheries to grow-out farms often takes place over long distances. However, transport restrictions during COVID-19 lockdowns have prevented buyers to reach fish hatcheries [25, 49]. Holding ready to sale fish fry as well as shrimp post-larvae for longer period with travel restrictions increases the risks of mortality and economic uncertainty [22, 38•]. A number of hatcheries in the USA have implemented new safety protocols with fully disinfected transport units during the pandemic [50].

Some aquaculture practices are often dependent on wild-caught fry, such as cod, eel, grouper, milkfish, prawn, shrimp, and tuna [51–53]. The collection of wild fry has been reduced due to the pandemic-related transport restrictions, lockdowns, and social distancing. The availability and supply of wild-caught prawn and shrimp post-larvae in Bangladesh have been considerably reduced during the pandemic [49]. The price of wild-caught shrimp post-larvae has also increased in Bangladesh [25]. Inadequate supply and high price of wild post-larvae has increased the price of hatchery-produced post-larvae, and thus, many farmers in Bangladesh and India have reduced stocking of shrimp post-larvae in their farms [37, 38•, 49].

### Feed and Feeding Challenges

Feed is one of the most essential inputs in aquaculture. There is great potential to increase farm productivity through increase feed supply at certain level. Various feeds are used for aquaculture, including industrially manufactured pelleted feed, farm-made feed, and supplementary feed. Large-scale commercial aquaculture is primarily dependent on industrially manufactured pelleted feed, while farm-made and supplementary feed are used by small and medium farmers [54]. Based on the size of stocked fish, feeds are categorized into nursery, starter, grower, and finisher, with floating and sinking types. A variety of ingredients are used in feed manufacturing, including fishmeal, fish oil, soybean oil, oilcake, rice bran, wheat flour, maize, meat and bone meal, calcium phosphate, salt, and vitamin premixes [54].

In most Asian aquaculture countries, feeds are usually manufactured domestically while some feed ingredients are imported. However, there is great concern of feed supply, availability, access, and limit feed application in grow-out operation during the pandemic. The availability and supply of feed ingredients have been affected by border closures and restrictions of cargo movements [5•]. Transport restrictions, inadequate logistic support, and quarantine measures have impeded feed supply. Importing feed ingredients and procurement of raw materials by feed companies have also been affected by COVID-19 [48•]. Thus, the accessibility and availability of feed ingredients in local markets have decreased considerably during the pandemic [49]. In Bangladesh, feed price increased about 10–12% due to inadequate supply of raw materials, labor crisis, and increased transportation and operation costs to maintain health guidelines [25]. Pandemic-related increased feed prices have affected aquaculture farmers in the Mekong region [26]. Locally available feed ingredients may help to cope with the pandemic crisis for aquaculture in Ghana [55]. Although many feed ingredients are sourced from locally available agricultural by-products (e.g., oilcake, rice bran, wheat bran), lockdowns and transport restrictions affect the supply of these ingredients in Bangladesh [49]. In fact, limit on the mobility of people has severely affected feed supply and transportation.

Many feed companies are unable to operate full range due to the shortage of feed ingredients. Some feed companies have been closed and unable to resume full-scale operation [21]. In Peru, the world’s largest fishmeal producer and one of the largest fish oil producers, was shut down due to national lockdowns [6]. Salmon farming needs a considerable amount of fishmeal and fish oil from wild-caught fish for feed production. However, wild fishing as well as capture fish production has been affected during the pandemic [56, 57]. Because of restrictions on banking services, many commercial feed manufacturers find difficulties to continue their operations. Some industrial feed companies are vulnerable to bankruptcy due to the COVID-19 pandemic [21, 58]. The pandemic crisis has also affected non-industrial feed producers in Bangladesh [21].

### Constraints in Agricultural, Chemical, and Pharmaceutical Input Supply

Aquaculture requires other inputs, including fertilizers, growth hormones, lime, antibiotics, and probiotics [59]. The application of fertilizers stimulates the growth of natural feeds (e.g., phytoplankton, zooplankton, benthos, and periphyton) in ponds and thereby increases fish yields. Although intensive aquaculture may not require fertilizer use, extensive and semi-intensive farmers apply fertilizers as a partial replacement for formulated feeds [60]. For example, organic (cow dung) and inorganic (triple super phosphate, urea) fertilizers are commonly used to enhance natural productivity in fish farms in a number of Asian countries [60]. Fertilizers are also used in earthen ponds for carp polyculture in Central and Eastern Europe [61].

Many fish farmers in aquaculture-producing countries also apply lime in fishponds to maintain a healthy and productive environment for preventing fish diseases and parasites. For example, lime is commonly used for shrimp culture in India, Thailand, and Vietnam to increase soil pH and kill unwanted organisms [62]. An increasing number of farmers in developing countries apply growth hormones and probiotics for increasing fish productivity [63, 64]. The microbial organisms used as probiotics are widely applied for carp, shrimp, and tilapia culture in China [65]. Antibiotics are also used in fish and shrimp farms in Vietnam for water purification, disinfection, fish health management, and disease treatment [66].

However, the availability and supply of these inputs in aquaculture have been affected by COVID-19–related transport restrictions and lockdowns. Interruption of chemical fertilizer production and distribution with logistical constraints affect aquaculture practices in Bangladesh, as farmers are unable to apply chemical fertilizers [67]. The supply of organic fertilizer, such as cow dung, may be reduced due to affecting livestock production by COVID-19 [68, 69]. Supply chain disruptions and transport restrictions also affect lime and probiotic application in aquaculture. The supply of medicines in aquaculture has also been affected by border closures and restrictions of cargo movements [5•, 67].

## Farm to Plate: Disruption in Fish Supply

There are concerns of aquaculture products by hindering fish supply from farmers to consumers. The COVID-19 outbreak has had great impacts on seafood supply chains [70]. The protection measures against the spread of COVID-19 have directly affected each step of fish harvesting, marketing, and exporting that may undermine the economic viability of aquaculture (Table 1). Globally, the aquaculture sector has been jeopardized by COVID-19, leading to various consequences, including seafood markets [71]. The COVID-19 crisis is having a series of adverse impacts on aquaculture output market chains. Ultimately, the pandemic lockdowns with domestic and international transport interruptions and travel restrictions have led to reduced capacity in marketing of aquaculture products.

### Concern of Fish Harvesting and Farmgate Price

Fish harvesting in aquaculture has been affected much by the COVID-19 pandemic, as producers cannot sell their fish due to market disruptions. Following COVID-19 restrictions, many fish producers have been unable to sell their products and, thus, maintain vast quantities of live fish that may increase production costs [5•]. There is a risk of aquaculture production during the pandemic due to not harvesting fish on timely as a result of lower seafood demand in markets [4••, 7••]. If there is no fish harvesting during the pandemic, fish in ponds means continue feeding which consists of high production costs with increased risks of fish mortalities and uncertainty of economic profitability [7••]. Shellfish farmers and traders in China have experienced a decline in profits during the pandemic [72]. Pangasius and tilapia farmers in Bangladesh were potentially putting them in debt [58].

The COVID-19 pandemic has affected price for farmed products, as the price of fish and shrimp has decreased in many countries [48•]. Aquaculture farmgate prices were consistently lower than average in many African and Asian countries during 2020 [44]. It has been reported that shrimp price in Bangladesh has reduced by 20–35% during the pandemic [25]. The price of prawn in the Philippines has decreased as much as 50% during the pandemic [73]. In Vietnam, the average farmgate price for pangasius was around US$0.78 per kg in 2020, below the average breakeven level [34]. Comparatively larger fish farms in the Mekong region (e.g., Cambodia, Laos, Myanmar, Thailand, and Vietnam) have experienced more negative impacts from lower fish market prices than small farms [26]. Many farmers in Bangladesh delayed their fish harvests to cope with a low market price of fish [49]. Uncertainty over prices and considerable price drops can be challenging for the aquaculture sector [6].

Farmers and traders in most aquaculture-producing countries have experienced reducing fish supply with domestic sales due to concerns about harvesting and farmgate prices. Fish landings and distribution have dropped in many aquaculture-producing countries, including Bangladesh, Egypt, India, Myanmar, Nigeria, and Timor-Leste as a result of national and regional lockdowns [48•]. In the EU aquaculture sector, the pandemic has led to a 17% decrease in sales volume and an 8% decrease in prices [32]. Declines in supply and demand for fish with uncertainty of COVID-19 trend may have adverse effects on future aquaculture production [6].

### Constraints in Fish Marketing

**Fig. 3:**
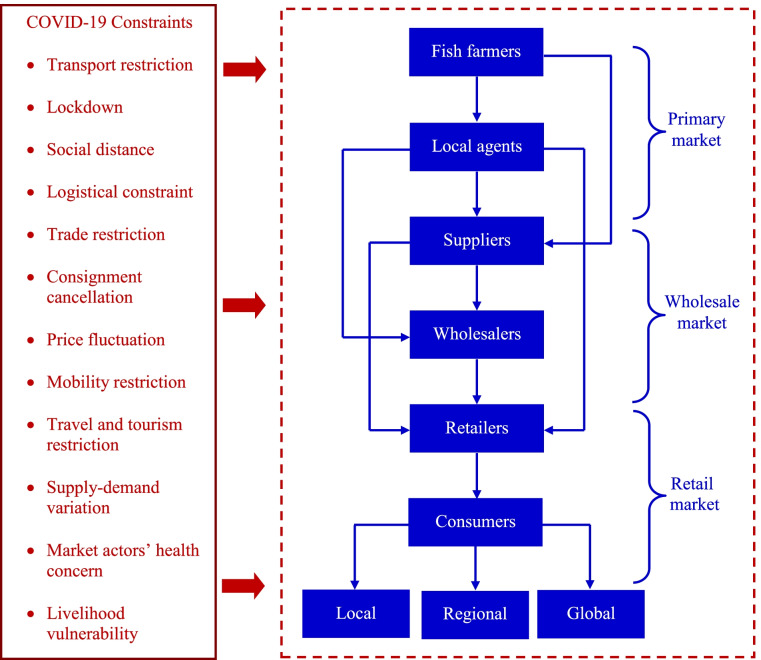
Fish marketing has been affected by the COVID-19 pandemic

Sustainable aquaculture production relies on fish marketing with economic returns. Globally, fish is the most highly traded food product [9]. Fish marketing is important to connect farmers and consumers for contributing significantly to the value-adding process. The demand for fish is usually high in markets, and thus, a strong network has developed with intermediaries and traders intervening between farmers and consumers. However, fish marketing activities have been affected much by the COVID-19 crisis (Fig. 3). Globally, seafood market disruption caused by the pandemic has already created problems in the aquaculture sector. Concerns arise of seafood marketing by the pandemic, which has been seriously impacted economic loss [6].

The COVID-19 crisis has been dramatically affected seafood marketing due to the public health risk of contamination, which is restricted fish trading [74]. Many wholesale and retail fish markets in developing countries were crowded and congested during the early stage of the pandemic, presenting health risks to traders and consumers [5•]. Thus, most wholesale, retail, and open fresh seafood markets were interrupted, banned, or stopped during the pandemic. Because of social distancing and confinement measures, the disruption of fish marketing channel occurred that have led to closure of many fish markets, globally [6]. The restriction of domestic transport and internal travel is likely limiting the trading and marketing of aquaculture products [4••]. The availability of and access to fish in local markets has also been severely affected owing to lockdowns and transport restrictions.

Because of the pandemic, interruption of wet fish markets has affected fish distribution and sale. A decline in access to raw fish with a reduced number of buyers may have decreased to reduce sale of fish [48•]. Thus, demand for raw fish in wet markets has been dropped. The drop of seafood demand with the complexity of the supply chains due to perishable products with labor-intensive activities has made the operation of seafood marketing loss-making [75].

### Challenges in Seafood Exporting

Seafood exporting is a global value chain, which has been declined along with demand, as the pandemic has severely affected seafood shipment [15••, 76]. Seafood exporting is traditionally involved in shipping products to overseas markets. However, restrictions on port access, transshipment, and maritime transportation during the pandemic has greatly affected seafood exporting [77, 78]. Moreover, seafood exports have declined due to the cancellation of trade events and discontinued shipment by buyers [7••, 20, 79].

Seafood grown in aquaculture for export market has been seriously affected by the disruption of international transport [5•]. Transport and cargo movement restrictions have significantly reduced imports of aquaculture products [4••]. High-value commodities in aquaculture has been negatively affected due to reducing export as a result of flight closures and massive drop in sales [4••]. With transportation cutoff in most aquaculture-producing countries, there have been fewer export buyers. In April 2020, seafood imports by the EU were a sharp declined by 15% [80]. In Galicia, Spain, the volume of seafood exports and imports decreased by 10.3% and 6.6%, respectively in 2020 due to disruption of international markets, particularly France, Italy, and Portugal [81].

Fish processing and exporting companies have been affected during the pandemic. Layoff staff in fish processing and exporting companies has also affected seafood trade. Usually, a large number of workers are involved in seafood processing plants, and thus, resuming processing activities with the same number of workers is logistically challenging due to maintaining physical distance [6]. Fish processing has also been affected by worker shortage due to COVID-19 illness, 14-day quarantine process, and travel barriers for seasonal or migrant workers [7••, 78]. Limits on the mobility of people due to lockdowns have affected the provision of seafood quality, safety, and certification checks for international markets. The risk of bankruptcy has also increased for seafood processing and export companies during the pandemic [58, 75].

The economic effects of the COVID-19 pandemic on aquaculture production could have severe consequences for the exporting of seafood. For the first quarter of 2020, lost sales of aquaculture products in the USA were estimated to be over US$1 million [82]. In India, shrimp exporting may be declining by 40% in the current season [38•]. Fish export from China decreased by 15% in 2020 due to the impacts of COVID-19 pandemic on fish trade [83]. Tilapia export from China has already been affected by the COVID-19 [84]. Cancellation of orders by overseas buyers has affected crab and shrimp export from Bangladesh [25, 47]. Total export value of Vietnamese pangasius dropped by 29% in the first 9 months of 2020 than the same period in 2019 [34]. However, seafood exports from Vietnam overcome the pandemic in 2021, as the total export value of pangasius has increased by 8.4% compared to 2020 [85]. Caution is still needed despite the brighter outlook. One of the policy mechanisms to adjust the pandemic is to reduce export sale while increasing domestic sale. To cope with the COVID-19 crisis, the Vietnamese authority has called for increased emphasis on domestic market for pangasius that may absorb 10–20% of production [34]. Nevertheless, changes in domestic demand for fish affect storage, resulting in increased food waste and loss [5•]. Thus, appropriate policies should be developed to maintain the balance between domestic and export market demands.

## Impacts on Livelihoods

About 10% of the global population depends directly or indirectly on aquaculture and fisheries for their livelihoods [9]. Globally, the pandemic has affected the livelihoods of millions of households relying on aquaculture for their income [7••, 26]. The pandemic has dramatically endangered the livelihoods of aquaculture farmers and associated groups, with high socioeconomic vulnerability, by reducing income of farming households, input suppliers, value chain performers, and market actors [7••, 37, 86]. The livelihoods of various supply chain actors in the aquaculture sector have been affected by the COVID-19 pandemic. In India, about 1.2 million people are employed in the shrimp sector, and the pandemic has affected the livelihoods of many supply chain actors [38•]. The livelihoods of suppliers, transporters, and day laborers in several aquaculture-producing countries have faced difficulties [48•]. Thus, the income of supply chain actors has been affected during the pandemic and subsequently impacts socioeconomic conditions.

A range of associated groups in a network of fry supply, such as hatchery operators, fry traders, intermediaries, transporters, and day laborers, has been affected by the COVID-19 pandemic. The livelihoods of people associated with fish hatcheries are also vulnerable due to stopping hatchery operation, and thus, many people have been unemployed during the pandemic [47]. Because of pandemic-related restrictions on banking services, many hatchery operators and related workers find difficulties to involve in their tasks. Aquaculture-related enterprises, such as fish hatcheries, are vulnerable to bankruptcy as a result of the COVID-19 pandemic [21].

The livelihoods of people in fish feed companies, particularly workers and day laborers, are vulnerable because of limited operation or not operating full scale during the pandemic. The COVID-19 pandemic has also affected the livelihoods of people in fish feed marketing [21]. Transport restrictions, lockdowns, and physical distancing have prevented buyers to reach feed industries. Thus, the livelihoods of people in feed marketing channel, such as suppliers, transporters, wholesalers, retailers, intermediaries, and day laborers, have been affected by the pandemic. In fact, market chain actors as well as suppliers have faced difficulties to find job as the feed companies have faced difficulties to operate full range during the pandemic [21].

Mobility in seafood marketing systems is important in terms of fish supply, income, and employment generation around the world. A large number of people are usually involved in diverse livelihoods through seafood supply chains, including fish transporting, sorting, grading, icing, processing, and marketing. However, the livelihoods of a certain group of people in seafood marketing have been affected during the pandemic [5•, 6, 15••, 82]. Fish market actors, including wholesalers, retailers, suppliers, and transporters, have been unemployed during the COVID-19 crisis. Financial support and institutional livelihood assistance (e.g., cooperative society, farmers’ organization, market access) have been suggested for resilience during the pandemic [73].

Globally, women account for just 14% of the 59.51 million people primarily engaged in aquaculture and fisheries, and their involvement in aquaculture is higher than that in fisheries [9]. In the seafood sector, women comprise 15% of the harvesting workforce and 80–90% in seafood processing [87]. During the pandemic, the employment status of women in the seafood sector appears to have declined more than men due to impacts on processing, trading, and wet markets, where women were heavily involved [44]. Female participants in the aquaculture sector are likely to be hit harder than men by the adverse impacts of COVID-19 pandemic due to unemployment, reducing income, gender inequality, and household responsibilities [87].

## Affect Seafood Consumption

The seafood industry globally undermines due to the onset of COVID-19 pandemic that threaten food and nutrition security [15••]. Seafood consumption is disproportionately affected during the pandemic. Reducing seafood production, supply disruptions, and affecting market prices have also affected consumption patterns. Moreover, reducing employment and lower incomes during the pandemic have affected seafood consumption in the world [88]. Globally, per capita fish intake fell to multi-year lows at 19.8 kg in 2020 from 20.5 kg in 2018 due to the pandemic [12].

Following lockdowns and transport restrictions, the pandemic has reduced domestic fish demand and consumption. Demand for seafood in rural and urban communities in many developing countries has decreased as a result of COVID-19–related unemployment [21]. Moreover, domestic fish consumption has declined due to reduce income of urban and city dwellers during the pandemic. Before COVID-19, 87% of households surveyed in Dhaka, Bangladesh, consumed fish at least six times a week, which dropped to 37% during the early phase of the pandemic [89]. At the same time, retailers sold on average 45% less fish in India, indicating a dramatic reduction in seafood consumption [44].

The cancellation of public and private programs and closure of catering services affect the demand of fish from aquaculture. The cancellation of Lunar New Year celebrations in China, which are traditionally associated with the consumption of high-value seafood, has had adverse impacts on seafood consumption [6]. In fact, the cancellation of various events and celebration programs with lockdowns and travel restrictions has had negative impacts on demand for high-value fish, such as mollusks (e.g., clam, mussel, oyster, scallop), salmon, seabass, seabream, and shrimp [6, 12].

As most countries implement lockdown measures across the world, the demand for seafood from hotels, motels, restaurants, and tourisms has been greatly reduced [7••, 90, 91]. Shellfish consumption is affected largely due to closure of tourisms, hotels, and restaurants [45]. The consumption of seafood has declined due to the closure of restaurants with the loss of restaurant sales [92–94]. The demand of seafood from catering, hospitality, entertaining guests, tourism, and various social programs has also considerably reduced because of lockdowns, physical distancing, and transport restrictions [94–96].

The consumption of seafood is important and, thus, must be advocated to ensure access to, and the availability of, healthy diets that address food insecurity and malnutrition [88]. Alternative seafood networks can be developed in response to the pandemic [76]. Because of limited or no access to hotels and restaurants during the pandemic, home cooking, home delivery, and takeaway food services through online platforms have been accelerated [97–100].

## Conclusions

The COVID-19 pandemic has adverse impacts on aquaculture value chains. The pandemic crisis has negatively affected aquaculture input supply. Because of inadequate and irregular supply of inputs, there is great concern of aquaculture practices, which may undermine future production. Globally, the aquaculture sector has been jeopardized by the COVID-19 crisis due to disruptions of input supply and output distribution. The pandemic has also severely disrupted the marketing of aquaculture products. The COVID-19 outbreak has had negative impacts on seafood harvesting, marketing, and exporting. Globally, the pandemic has also affected the livelihoods of millions of households in the aquaculture sector. Moreover, reducing employment and lower incomes during the pandemic have affected seafood consumption in the world.

To characterize the further impact of COVID-19 on aquaculture value chains, quantitative assessments are needed. Moreover, there is an urgent need for collaboration with major stakeholders to establish short and long value chains for sustainable aquaculture practices. Sustainable aquaculture production also needs greater attention from international agencies, donors, government and non-governmental organizations, researchers, and policymakers to better develop value chains of the aquaculture sector. Institutional support and key stakeholders’ collaborations are needed for policy formulation of sustainable aquaculture practices through establishing value chains. International and national financing institutions can provide support to build up value chains of the aquaculture sector. Financial assistance is one of the policy mechanisms to rebuild aquaculture value chains [7••, 58, 73]. Access to credit at reasonable interest rates would be essential for aquaculture farmers and associated groups to establish supply chains and value addition to seafood. Livelihood support (e.g., financial aid, social protection) in the aquaculture sector can also help to rebuild supply chains and seafood marketing.
